# The Uses of Ergot in Obstetrics*Read before the Buffalo Medical Club, Nov. 1, 1882.

**Published:** 1882-12

**Authors:** Chas. G. Stockton


BUFFALO

Medical and Surgical Journal.

VOL. XXII. DECEMBER, 1882. No. 5.

### Original Communications.

### THE USES OF ERGOT IN OBSTETRICS.*

BY CHAS. G. STOCKTON, M. D.

Of these uses of ergot, an agent lending the parturient woman new power, it would seem unnecessary to speak to the profession, which, for the past 75 years, has had the action of the drug under constant observation. But, recollecting that the cynicism of the times questions the uses of such great institutions as Christianity and Divine law, it is not so remarkable that it should have doubts concerning simples in their relation to physiological forces. Though it would seem that the action of ergot is well enough understood to assure its successful application and do away with such remarks as that made at the recent meeting of the American Gynecological Society by Dr. Joseph Tabor Johnson, of Washington, that he believed the human race would be better off without than with ergot in the management of labor, and that it certainly should never be given to a primipara. Such an expression is to be deplored, and is akin to the statement that vaccination is ineffectual, or that mercury is

* Read before the Buffalo Medical Club, Nov. 1, 1882.

useless in the treatmeut of syphilis; and, although it is a digression from the question, I must be allowed to state my humble opinion that our medical discussions are not so conservative as they should be—for, if so-called liberal views *do* lead to growth in medicine, such growth is apt to partake of the nature of neoplasm.

The manner of th- action of ergot is to some extent unsettled, but there is agreement that its administration is followed by vaso-motor spasm, and probably by contraction of all unstriped muscular tissue. In 1870, Holmes, after repeated experiments, concluded that the action of the drug was peripheral. After section of a sympathetic he found the vessels supplied by this nerve to contract after the introduction of ergot; and when the drug was injected into the right jugular, the arterial pressure at first fell, which our experimenter attributed to spasm of the pulmonic capillaries retarding the flow of blood into the left ventricle—and from these he inferred that the action was peripheral—and the inferences were supported by the experiments of Wernich in 1872.

But Carl Vogt, in 1869, and Eborty, in 1873, found no vaso-motor spasm in a part where the sympathetic had been cut, and they found no arterial depression to precede the rise, but, on the contrary, a prompt and powerful increase of arterial tension followed the injection of ergot. Dr. Geo. B. Wood mentions a case where it produced great prostration with almost absence of pulse. It is known that when ergot is applied to bleeding surfaces, it acts as a styptic; and Dr. Mundé says that oxytoxic effects are obtained by introducing it into the uterus in form of a suppository. In these instances it would seem to have a local and not a centric action. Without questioning *how*, there is less conflict in regard to its action upon uterine muscle; for most authorities say that it induces contraction in a certain proportion of cases. Wright found it to fail in this respect; so did Bonjean. But Dies, Percy, Laurent, and others, found it to cause abortion in various animals, and M. Bodin reports an epidemic among cows from feeding upon ergotized grasses. Wood says it will often act as an abortifacient; Rigby gives directions for using the secale for inducing premature labor, which, he says, rarely fails to produce the desired result.

Prof. Ramsbotham states that he has made a great number of trials and found that expulsive action soon followed its exhibition with very few exceptions. "Administered to pregnant women," says Phillips, "ergot excites uterine contraction in a manner truly remarkable, facilitating parturition or bringing on abortion." Says Ringer: "Its effects are most expressed upon the womb, especially when pregnant, exciting in the gravid uterus powerful and continuous contractions." Bartholow recommends it for expelling abnormal growths from the uterus by means of the uterine contractions thus excited, yet, on the subject of abortion, remarks: "It is no longer a matter of doubt that ergot promotes uterine contractions; that it originates them without previous efforts of the womb is questionable." Bartholow needs explaining. Dr. Frederick reports a case where ergot was successfully given for the induction of labor. A number of old practitioners I have questioned, and nearly all expressed belief in this property of the drug. During labor the fungus has been used very largely in every stage. Country accoucheurs, called from a distance to attend women whose labors are tedious, often give it without much regard to the stage, or the condition of the cervix, and, undoubtedly, have the satisfaction of hastening delivery in a great majority of cases. The experience of these men is very large. I knew a man who had attended over 1000 confinements, who seldom used forceps, who generally gave ergot, and whose reputation for good luck was enviable. We must remember that men have notions and are predetermined; and it will be well for us who habitually use forceps and lament the lacerated women and dead fœti of the ergot-giver, to compare the results of our cases with those of him who carries his spurred rye and leaves his instruments at home.

Junius, in one of his incomparable letters, remarks that the man who advocates a cause may begin with a weak faith and argue himself into a strong belief, and I am not sure but this principle is operating upon me in my zeal to present before you properly the obstetrical uses of ergot. Many give ergot in the first stage of labor when the os is not dilated, sometimes not dilatable—confessedly adventuresome practice, although frequently successful. It is advised to withhold the drug until these changes have taken place; then when the pains are not of sufficient strength, when there is a safe presentation, and when there are no deformities of the pelvis or soft parts, it should be given; and a good rule for action at such times seems to be that of a friend, who says that when the membranes are pushed down, forming the caput succedaneum, or when the scalp is wrinkled during a pain, ergot should not be given, as the pressure is great enough for the head to bear, and if assistance is needed, forceps should be applied; but when the pains are too feeble to produce these signs, ergot is indicated and should be given in 20-drop doses every 20 to 30 minutes until the contraction, are sufficient, and should then be discontinued, to be again given when the pains become weak. It may be said that this will bring about unremitting tonic contractions. This is not always the result if the rye is given in small doses and judiciously, and as for that matter, there are cases of natural labor where only one continuous, enormous contraction is had from the first intimation of labor to the birth of the child. I have reports of two such cases.

In placenta praevia ergot is often given with benefit, and in partial placenta praevia its use is highly recommended with the expectation of producing firm contraction, and, from the pressure of the fœtal head upon the placenta, prevent hemorrhage. It is proper to use the drug in such cases during the first stage, upon the authority of Ramsbotham, Lusk and Barker. If the os is not well dilated it should be, and ergot still given, is the opinion of Dr. Hanks, of New York.

In accidental hemorrhage it should be given in moderate doses, and Rochester says it will sometimes prevent impending abortion; but when the hemorrhage is excessive, Playfair advises the rupture of the membranes and the production of firm contraction by full doses of ergot. In breech presentations, when the breech has reached the plane of the pelvis, Trousseau says that ergot should be given, and the idea appears to be a good one.

My opportunities for hearing medical lectures, a method of teaching which Morell Mackenzie pronounces a thing of the past, have been limited, but with all the future has in store for me, I am sure I shall never forget that graphic description of post partum hemorrhage which the late Jas. P. White was accustomed to give us in his lecture on that subject, nor shall I forget with what vigor be pointed to ergot and its uses, when the hour of the "kite-tailed tampon" had passed away. Everybody gives ergot in post partum hemorrhage, and if the drug had no other uses we ought all to unite in praises to Ceres that every kernel doesn't go on to full fruition, and that we have the fungus, secale cornutum.

To excite uterine contractions and induce abortion or labor; to hasten the termination of the first stage when the os is dilatable; to expedite the second stage, and, in its complications, add to the safety of mother and child; to prevent hemorrhage in abortion from the first month to the last; to facilitate the discharge of the secundines; to procure tonic contraction after the escape of the placenta and thus avoid atony of the uterus and post partum hemorrhage; to control flooding; to speedily check relaxation of the uterus, intermitting with distressing bearing-down pains and hemorrhage; to prevent, by means of early tonic contraction, after-pains in general, particularly in women who have borne many children; to prevent absorption of septic material, thus acting either prophylactically or curatively in puerperal septicæmia; to secure and hasten the process of involution after abortion or labor at full term; to relieve long-continued lochial discharges, is ergot useful, in some cases indispensable.

---

